# Research about DoS Attack against ICPS

**DOI:** 10.3390/s19071542

**Published:** 2019-03-29

**Authors:** Jianlei Gao, Senchun Chai, Baihai Zhang, Yuanqing Xia

**Affiliations:** School of Automation, Beijing Institute of Technology, Beijing 100081, China; jianleixinye@163.com (J.G.); smczhang@bit.edu.cn (B.Z.); xia_yuanqing@163.net (Y.X.)

**Keywords:** DoS attack, industrial cyber-physical system (ICPS), security zones, mimicry security switch strategy

## Abstract

This paper studies denial-of-services (DoS) attacks against industrial cyber-physical systems (ICPSs) for which we built a proper ICPS model and attack model. According to the impact of different attack rates on systems, instead of directly studying the time delay caused by the attacks some security zones are identified, which display how a DoS attack destroys the stable status of the ICPS. Research on security zone division is consistent with the fact that ICPSs’ communication devices actually have some capacity for large network traffic. The research on DoS attacks’ impacts on ICPSs by studying their operation conditions in different security zones is simplified further. Then, a detection method and a mimicry security switch strategy are proposed to defend against malicious DoS attacks and bring the ICPS under attack back to normal. Lastly, practical implementation experiments have been carried out to illustrate the effectiveness and efficiency of the method we propose.

## 1. Introduction

A cyber-physical system (CPS) is a physical system that combines physical plants with network systems for data transmission and control functions, which has attracted worldwide attention after it was put forward in 2006 by the U.S. National Natural Science Foundation [1]. The CPS usually integrates some physical processes, data communication capabilities, sensors, data calculation and process control. It utilizes computers and networks to monitor physical process and control production parameters. It realizes combined calculations with physical processes depending on the real-time data interaction. CPSs are ubiquitous in modern life, ranging from current sweeping robots to global energy power system networks, which include smart cities, medical systems, military command systems, etc. 

There are a lot of different types of CPSs, whose most typical application is the industrial control system (ICS), also called industrial cyber-physical system (ICPS), such as a supervisory control and data acquisition (SCADA) system, or programmable logic controller (PLC) system. They are widely used in a variety of industries, especially those related to critical national infrastructures, such as smart grids, energy production and transmission, smart cities, municipal engineering, the petrochemical industry and so on [2].

In recent decades, the corresponding technology has been developed dramatically. In order to enhance the facilities, reduce the complexity and cut down costs, more and more ICPSs are being upgraded with the latest communication and control technology, such as network communication, wireless sensors networks, multi-agent systems and so on. Generally speaking, ICPSs with integration modern cyber-technologies, which include Internet technology, cloud technology, Internet of Things and so on, have been using those technologies to communicate with each part, monitor plants and control physical processes. However, network attacks and vulnerabilities that have produced great risks and a large number of information incidents have been triggered due to the open networks protocols, which have already resulted in serious damage.

In recent years, many ICPS incidents have happened around the world. In 2010, the Iranian nuclear incident where the country’s nuclear energy program was attacked by the “Stunex Virus”and “Duqu Trojan” that was detected in many countries in 2011, was considered the first premeditated destruction aimed at critical ICPSs. In 2012 some security experts found that the “Flame virus” not only attacked Iran, but also affected the entire Middle East region. A German steelworks suffered from a cyber-attack, which resulted in the control systems and production systems being forced to stop in 2014, but the most striking example was the collapse of the Ukrainian electricity grid in December, 2016.

**Figure 1:**
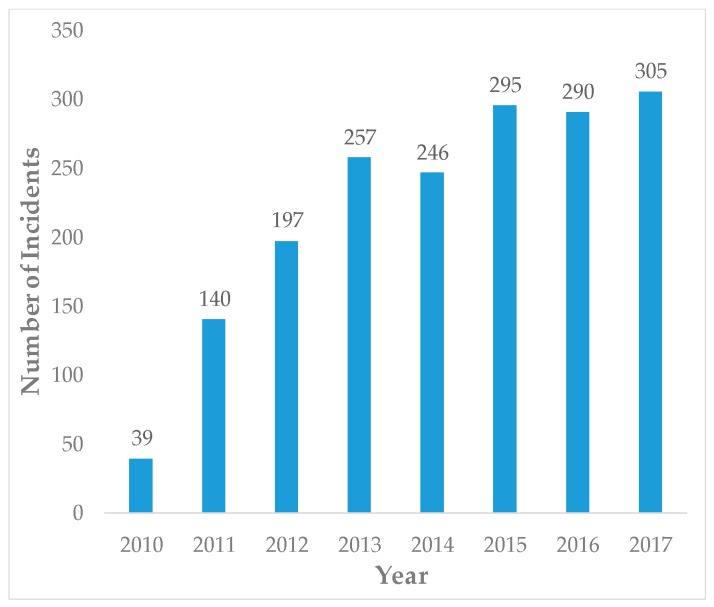
ICPS security incidents by year [3].

The report [3] published by ICS-CERT of China provides statistics of ICS information security incidents from the Industrial Control Systems Cyber Emergency Response Team (ICS-CERT), which shows that more and more ICPSs are being attacked by various malicious cyber actions as depicted in Figure 1. As is seen from the chart, obviously the incident occurrence is on the rise. 

The ICPSs, especially the control systems of ICPSs, are connected to the public Internet, which raises greater security challenges than pure information systems when they are under cyberattack. As ICPSs are media to bridge physical process and virtual world of information, so the availability of data, including control data and communication data, is more important because of its effect on real-time performance. That is to say, once an ICPS is attacked by malicious attacks, it will cause more serious consequences than attacks on pure information systems.

The rest of paper is organized as follows: in Section 2, some related works are presented. Section 3 discusses some basic knowledge and mathematic models, which include an ICPS model, DoS attack model, DoS attack effect on ICPS model and so on. The detection model and defense strategy are designed in Section 4. We present some experimental results and analyses in the following Section 5. The last part presents the conclusions.

## 2. Related Work

**Figure 2:**
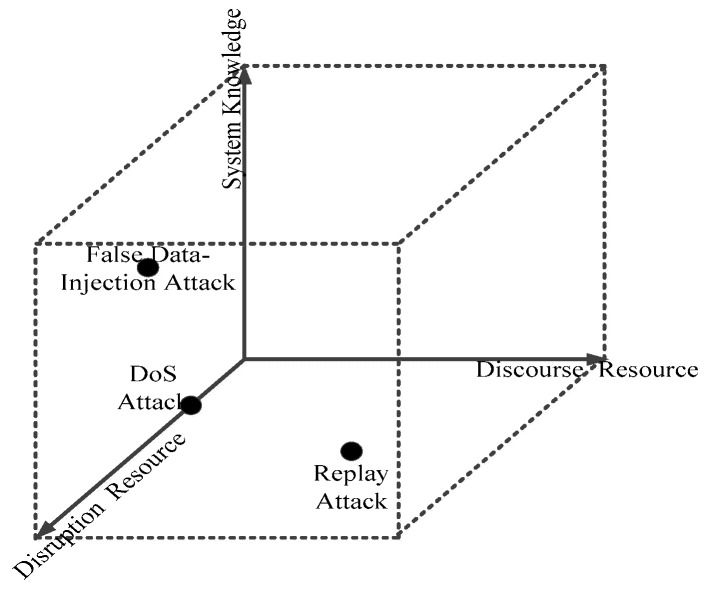
Three-dimensional attack space.

As is known to all, unlike traditional information security where more attention is paid to the protection of data, cyber-attacks on CPSs’ control networks usually wreck physical processes because of the existence of feedback networks, so the research and analysis on CPS must take both the cyber area and physical parts into consideration. Currently, there are various modes of attack against CPS, such as denial-of-service (DoS) attacks [4,5], bias injection attacks [6,7,8], zero dynamics attacks [9], convert attacks [10], zero response attacks [9], eavesdropping attacks [10] and so on. According to reference [10], the authors created a three-dimensional space, illustrated in Figure 2, to quantify them.

There have been a great deal of algorithms designed to analyze and solve these malicious attacks in CPSs [7,10,11,12,13,14]. They typically provide an explanation, system model and analysis, and control system experiments against different attack ways [10]. In [15], the authors do a lot of work about cyber-physical systems, supply a mathematical framework of the systems’ attacks and monitors, present some fundamental monitoring limitations from a system-theoretic and graph-theoretic perspective, and design a distributed attack detector and identification monitors. Reference [12] studies a general convex optimization method of estimation which demonstrates generic sufficient and necessary conditions instead of specific estimators. The current detection methods against cyber-attacks are based on statistical learning algorithms which could cause misleading alarms. Reference [13] adopts a mulit-order Markov chain framework based on supervised statistical learning to solve the above shortcoming. Besides, it designs an optimal attack strategy to destroy wireless sensor network control systems and worsens the cost function to maximum value and find a coping strategy in this way [14]. Among these efforts aimed at studying specific malicious attacks, the DoS attacks (including DDoS) has been widely studied because of their easiest implementation, most serious consequences and least system knowledge that is needed to destroy the communication channel between a system’s parts.

Many people have been devoted to studying DoS attacks against CPSs (including ICPSs), whose focus can mainly be divided into two parts: information security and control science. From the viewpoint of computer information security, people usually design an intrusion detection system (IDS) to protect targets from DoS attacks. The most typical methods are based on machine learning algorithms. For example: [16] proposes a new method based on support vector machine (SVM) which is motivated by the fact that the cloud environment is changeable/dynamic to detect DoS attacks. However, current IDS can’t detect two specific hardware Trojans (HT)-assisted DoS attacks (sinkhole and blackhole attacks) which is explained by quantifying the effects of attacks as packet loss rates [17]. In order to deal with this problem in embedded systems designed with Multiprocessor-System-on-Chip (MPSoC) architectures, the utilization of pipelined MPSoC (PMPSoCs) is selected and improved to detect DoS attack-based hardware Trojan attacks [18]. Although all the studies provide some reasonable and useful methods to prevent, detect, defend and eliminate DoS attacks, they all have their limitations and deficiencies, especially in dealing with the carefully designed network DoS packets. What is worse is that they all don’t consider the impact of attack on the physical part when they only fix their attention on the cyber layer. However, the physical part of ICPS is especially in need of strong real-time control data.

Many people study DoS attacks on ICPSs from the consideration of control theory. Some attack models and scenarios are given by reference [4] whose analyses are shown in Figure 2. Reference [5] uses the Tennessee Eastman challenge process to study the DoS attack through modeling the problem of DoS attacks as optimal stopping problems, which cause a change of the timing parameter in a physical process. The authors [14] analyze the problem of DoS attacks from the viewpoint of an attacker to study the optimal DoS attack strategy which can maximize the cost function of the linear quadratic regulator (LQR) controller. What’ more, Yuan et al. [19] use a unified game theory to improve the robustness by designing a resilient control network system. Obviously, these algorithms can validly address the influences of attacks against physical layer such as control systems, but most of them lack any study on the cyber layer, which cannot eliminate DoS attacks.

In this paper, we try our best to combine the two aspects of ICPS security research to detect and eliminate DoS attacks against networks, and effectively solve the impacts of the attacks in the physical area to maintain ICPSs’ normal operation. 

The main contribution of this work are: (1) we try to combine and information security method with a control theory method to study the DoS attacks against industrial cyber-physical systems (ICPSs), and propose a mathematical model of DoS attacks with a detailed explanation; (2) according to the influence of different attack rates against ICPSs, we study the time delay caused by attacks dividing the ICPS into security zones instead of studying it directly, which displays how a DoS attack destroys the stable status of the ICPS; (3) a detection method and a mimicry security switch strategy are proposed to defend against this malicious DoS attack and bring the abnormal operation of ICPS back to a normal status; (4) a practical implementation has been carried out to illustrate the effectiveness and efficiency of the proposed method, which gives us an inspiration to protect our critical ICPSs with multiple sets of redundant sub-control systems.

## 3. Preliminary Knowledge

### 3.1. ICPS Structure

**Figure 3:**
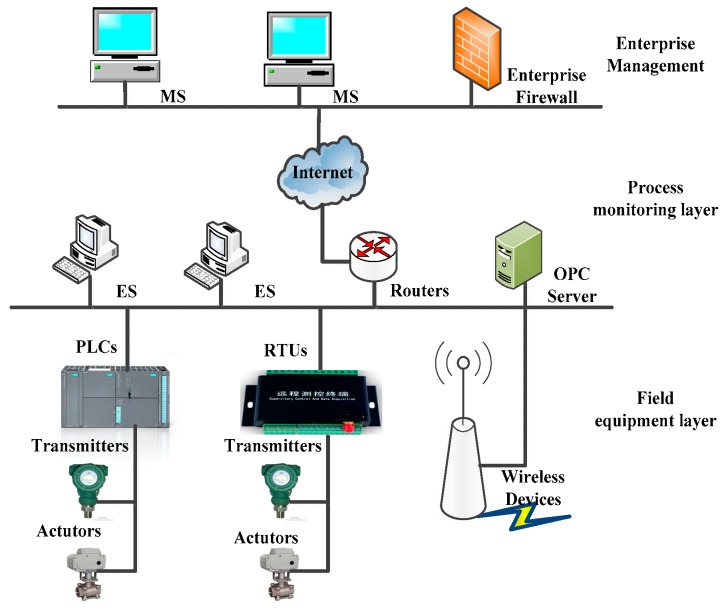
The structure of an ICPS.

With the improvement of information technology, more and more ICPSs adopt Ethernet technology based on the TCP/IP protocol, which makes the control system more integrated, improves information transfer rate and the compatibility between different systems and enhances the range of application. A typical ICPS structure is shown in Figure 3.

### 3.2. ICPS Model

Consider the following ICPS with multi-subsystems:

(1) $$\{\begin{array}{c} g=g(o_{i,})\\ o_{i}=o_{i}(p_{i},f_{i}) \end{array}\;(i=1,2,\cdots ,N)$$

where *i* is the index of sub-control system, *o_i_* is the *i*-th-sub-system, *N* is the sum of sub-control systems, *p_i_* is the transfer function, *f_i_* is the network characteristic function for which a detailed explanation will be given below. Consider the following physical system which is assumed a continuous linear dynamic system:

(2) $$p_{i}(x)=\{\begin{array}{c} \dot{x}(t)=A_{s}x(t)+B_{s}u(t)\\ y(t)=C_{s}x(t)+D_{s}u(t) \end{array}$$

where $x(t)\in {\mathbb{R}}^{n}$ and $y(t)\in {\mathbb{R}}^{m}$ show the system states and system output, respectively, at time t∈ℕ. Besides, the matrix of $A_{s}$, $B_{s}$, $C_{s}$ and $D_{s}$ are constant matrixes with related ranks.

**Assumption 1.**  *Only one sub-system o_i_ is running at one moment, and other sub-systems* $o_{j}(j\neq i)$ *are listening and in standby mode at the same time.*

### 3.3. DoS Attack Model

In this subsection, in order to build a DoS attack model, we need to provide some assumptions and a definition firstly:

**Assumption 2.**  *The time delay caused by the network’s background is not considerable.*

**Assumption 3.**  *The ICPS has more than one sub-system*$f=\{f_{i}|i=1,2\cdots ,m\}$*(m is the sum of sub-system), and each of them has different network parameters which include an IP address (*$l_{1}$*) and communication port (*$l_{2}$).

**Definition 1.**  *There exists an attack function*$a=\{a_{i}|i=1,2,\cdots ,m\}$*(m is the sum of sub-system), an attack operator*⊗*and attack set*$I_{H}=\{0,1\}$.

A Denial-of-Service (DoS) attack is defined as a means to send lots of network data packets to targets, which will shut down users’ computers and make the paralyze the communication network. DoS attacks accomplish this by flooding the target with traffic, or sending malicious information that triggers a crash. However, explicit DoS attack models are not given in [20], which lacks real network information, so we will define an attack model to explain how a DoS attacks a system.

According to Assumption 3, the attack function $a=\{a_{i}|i=1,2,\cdots ,m\}$ and the attack object (the ICPS with one running subject) have two parameters, we can get $a_{i}=a_{i}(l_{1},l_{2})$ and $f_{i}=f_{i}(l_{1},l_{2})$.

Applying Definition 1 and Equation (1), it can be obtained that:

(3) $$I_{H}=a_{i}⊗f_{j}=a_{i}⊗o(f_{i},*)$$

**Theorem 1.**  *Consider a DoS attack against ICPS, the attack* $a_{i}$ *is independent of each other and the attack object* $f_{i}$ *is independent of each other. Therefore, the attack set* $I_{H}=\{0,1\}$ *has:*

(4) $$\{\begin{array}{c} I_{H}=a_{i}⊗o(f_{j},*)=a_{i}⊗f_{j}=1,i=j\\ I_{H}=a_{i}⊗o(f_{j},*)=a_{i}⊗f_{j}=0\;,i\neq j \end{array}$$

### 3.4. DoS Attack Effect on ICPS

As we all know, a DoS attack will affect a system’s normal operation. As for how it affects the system, modelling system service performance from an information security perspective is relatively plausible in traditional information systems but not reasonable in an ICPS without consideration of stability of its physical parts. Studies in control science suggest the DoS attacks can increase the delay of control processes, and thus this will degrade the performance of the control system which is lacking details. Therefore, we try our best to explain how a DoS attack affects an ICPS’s performance with Definition 2.

**Definition 2.**  *We define a packets rate function*fr:

(5) $$fr=\frac{sum\;(packet)}{sum\;(time)}$$

Actually, the DoS attack destroys the system’s performance by increasing the time delay and this undoubtedly reduces the real-time control performance when the DoS attack lasts for a certain period. According to the network’s features and working principle, for a more intuitive explanation, we define a time delay *τ*, dangerous zone Ω and their relation with *fr* to explain the details here.

**Figure 4:**
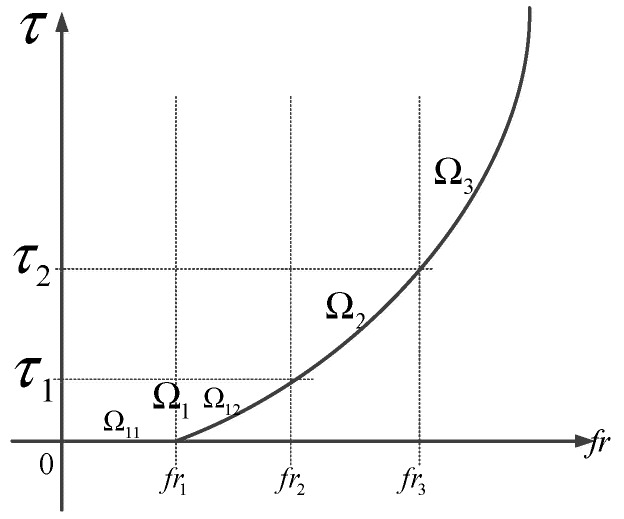
Relation between attack rate and time delay.

**Definition 3:**  *The ICPS has running zones*$\Omega =\left\{\Omega_{i}|i=1,2,3,4\right\}$*, and*$\Omega_{ICPS}$*represents the current running zone, which is shown in Figure 4*.

(6) $$S=\{\begin{array}{l} \Omega_{11},\;0\leq fr<fr_{1}\\ \Omega_{12}\;,\;fr_{1}\leq fr<fr_{2}\\ \Omega_{2}\;,\;fr_{2}\leq fr<fr_{3}\\ \Omega_{3}\;,\;fr_{3}\leq fr \end{array}$$

where $\Omega_{11}$ is an absolutely secure zone which indicates that no attack can affect the system’s normal operation. That is to say no attack is launched. $\Omega_{12}$ is a related security zone which indicates the system can still operate normally under attack. $\Omega_{2}$ is a hazardous zone which indicates the system runs abnormally under attack but does not crash. $\Omega_{3}$ is an absolutely hazardous zone which indicates the system has collapsed under attack. $\tau_{1}$ is the resilience time delay which means this time delay can be accommodated by the network under attack and does not have any negative effects. $\tau_{2}$ is the maximum time delay that the system can sustain. $\tau_{num}$ is the sum of the delays in the current communication network.

Merging $\Omega_{11}$ and $\Omega_{12}$, we can get:

(7) $$\Omega =\{\begin{array}{l} \Omega_{1}\;,\;0\leq fr<fr_{2}\\ \Omega_{2}\;,\;fr_{2}\leq fr<fr_{3}\\ \Omega_{3}\;,\;fr_{3}\leq fr \end{array}$$

From the above, it is known that $\Omega_{1}$ is a security area; $\Omega_{2}$ is a transient-normal area; $\Omega_{3}$ is an abnormal operation area.

**Explanation.**  *Nowadays, network devices are built with an inherent time delay, which does not affect the normal operation of the ICPS. Meanwhile, they also have an elasticity feature that has some capacity to bear a bit of large network traffic to maintain the normal operation of the ICPS. This is in conformity with the actual situation. Therefore, our assumption is reasonable and the experimental data provided later will prove it too.*

**Remark 1.**  *When the ICPS was attacked, every zone has following property:*

(8) $$\{\begin{array}{c} \overset{3}{\underset{i=1}{\cup }}\Omega_{i}=1\\ \overset{3}{\underset{i=1}{\cap }}\Omega_{i}=0 \end{array}$$

**Figure 5:**
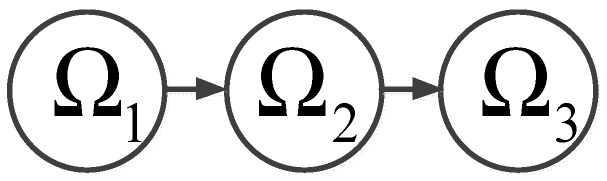
Attack zone migration process.

**Remark 2.**  *When the ICPS was attacked, every zone had the following migration process as is shown in Figure 5:*

**Theorem 2.**  *The necessary and sufficient conditions for the system to run normally are:*

*(1)*  $\Omega_{ICPS}\notin \Omega_{3}$ *and* $\Omega_{ICPS}\notin \Omega_{2}$
*(2)*  $\Omega_{ICPS}\in \Omega_{2}$ *but* $T_{ICPS}<\tau_{2}-\tau_{1}$

**Proof of Theorem 2.**  **Necessary condition:**

About condition (1), if $\Omega_{ICPS}\notin \Omega_{2}$ and $\Omega_{ICPS}\notin \Omega_{3}$ → $\Omega_{ICPS}\in \Omega_{1}$.

About condition (2), if $\Omega_{ICPS}\in \Omega_{2}$ but $T_{ICPS}\leq \tau_{2}-\tau_{1}$ → $\tau_{num}=0+\tau_{1}+T_{ICPS}<\tau_{2}$ → runs normally.

**Sufficient condition:**

About the proof of sufficient condition, we can use opposite to prove it. Firstly we assume the system is abnormal, so the time delay $\tau_{num}\geq \tau_{2}$.

Obviously, there are only two conditions which can satisfy it: $\Omega_{ICPS}\in \Omega_{3}$ or $\Omega_{ICPS}\in \Omega_{2}\;$ and $T_{ICPS}+\tau_{1}\geq \tau_{2}$

From the analysis of an attacker’s perspective, it is intended to implement the DoS attack plan that transforms the running zone $\Omega_{1}$ into $\Omega_{2}$, or even $\Omega_{3}$.

**Problem 3.1** **(Attackers’ Purpose)** (9) $$\{\begin{array}{c} \max\;T_{ICPS}\\ s.t.\;I_{H}=1 \end{array}$$

*The Problem 3.1 means that this malicious DoS attack is launched (* $I_{H}=1$ *) to increase the maximum communication time delay of data packets (* $T_{ICPS}$ *). According to the abovementioned Theorem 2, the increasing* $T_{ICPS}$ *to deteriorate ICPS’s normal running is equivalent to making the ICPS run in* $\Omega_{2}$ *and* $\Omega_{3}$ *, so the Problem 3.1 can be equal to:*

(10) $$\{\begin{array}{c} \Omega_{ICPS}\in (\Omega_{2}\cup \Omega_{3})\\ s.t.\;I_{H}=1 \end{array}$$

### 3.5. Mimicry Security Policy

**Assumption 4.**  *The time interval of switching between two different sub-systems is 0.*

**Figure 6:**
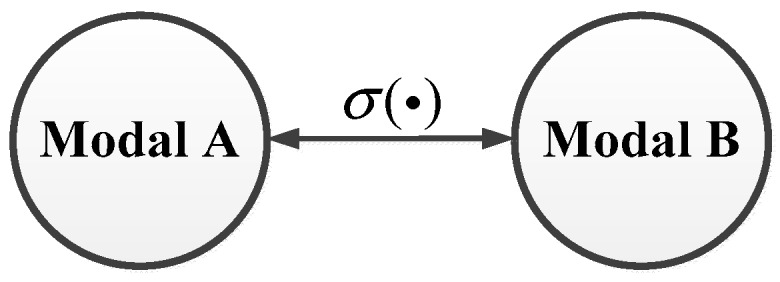
Mimicry policy.

In Nature a large number of creatures, the most typical example of which is the octopus, simulate other creatures through morphology, behavior and color, thus deceiving possible attackers and protecting themselves. This phenomenon is called mimicry, and it gives many living creatures a way to survive. Inspired by this ability, a large number of scholars began to study this mimetic defense strategy in the field of information security, and they have achieved excellent results. What’s more important is that this strategy is effective against many methods of network attack. Besides, the majority of important infrastructures have several sets of stand-by sub-systems, which provides good conditions for the application of this strategy, so we try to use a mimicry security policy to defend DoS attack against ICPS. First, we define a mimicry defense strategy σ(•), following modal transfer as is shown in Figure 6:

That means the ICPS with multi-sub-systems will change its running-sub-system which is the modality of the current moment using a mimicry defense strategy σ(•) when it is attacked by a DoS attack.

Replace σ(•) into ICPS system’s function:

$$\{\begin{array}{c} g=g(o_{i})\\ \sigma (•) \end{array}=g_{\sigma (•)}(o_{i})$$

Applying Equation (1):

$$g_{\sigma (•)}(o_{i})=\{\begin{array}{c} f_{\sigma (•)}\;(t)\\ p_{\sigma (•)}\;(t) \end{array}=\{\begin{array}{c} f{\;}_{\sigma (•)}\;(t)\\ \{\begin{array}{c} \dot{x}(t)=A_{\sigma (•)}x(t)+B_{\sigma (•)}u(t)\\ z(t)=\text{​}C_{\sigma (•)}x(t) \end{array} \end{array}$$

Adding the constraint condition attack set $I_{H}$, we can get the following Equation (11):

(11) $$\{\begin{array}{c} g_{\sigma (•)}(o_{i})\\ s.t\;I_{H}=1 \end{array}=\{\begin{array}{c} \{\begin{array}{c} f_{\sigma (•)}\;(t)\\ p_{\sigma (•)}\;(t) \end{array}\\ s.t\;I_{H} \end{array}=\{\begin{array}{c} \{\begin{array}{c} f_{\sigma (•)}\;(t)\\ \{\begin{array}{l} \dot{x}(t)=A_{\sigma (•)}x(t)+B_{\sigma (•)}u(t)\\ y(t)=\text{​}C_{\sigma (•)}x(t) \end{array} \end{array}\\ s.t\;I_{H} \end{array}$$

Equation (11) shows that the function of mimicry switch strategy is to change the sub-system of the ICPS under DoS (*I_H_*) to another sub-system to protect the ICPS. From the above analysis and assumption, we know that the cyber layer with a new network configuration has a natural immunity ability against DoS attacks after changing its sub-system using the mimicry defense strategy. These malicious attack packets cannot reach the ICPSS because of the new network configuration with a different IP address and port parameters. What’s more, the physical layer with a new sub-system which has a new control system has the capability to keep the system stable and reduce the time delay to ensure the real-time character after the ICPS changes its old model.

**Figure 7:**
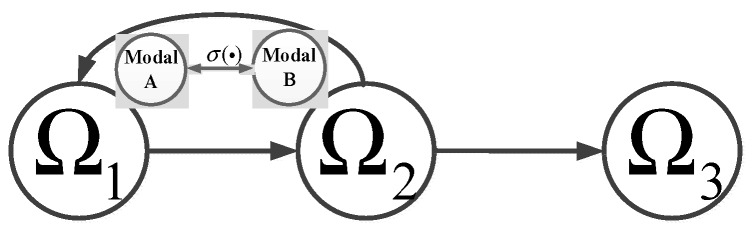
Zone transfer using mimicry strategy when under attack.

In short, the role of these security tactics is that ICPS’s running zone is transferred into a security zone from a transient-normal area as shown in Figure 7.

## 4. The Defense Strategy for DoS Attacks

In this section, some methods are developed to defend from DoS attacks, which contain a detection method and a mimicry security strategy to avoid the adverse effects of the attack.

### 4.1. The Detection of a DoS Attack

According to the physical system of ICPS shown in formula 2, we can get the model discretized by shift operator:

(12) $$\{\begin{array}{c} x_{k+1}=Ax_{k}+Bu_{k}+w_{k}\\ y_{k}=C_{k}+v_{k} \end{array}$$

where $x_{k}\in R^{n}$ is the *n*-dimensional vector of state variable at time k, $u(k)\in R^{m}$ is the *m-*dimensional system input vector at time k, $y_{k}^{T}$ is an *m*-dimensional observation vector at time k, $w_{k}\in R^{n}$ and $v_{k}\in R^{m}$ are measurable white noises whose means are 0 at time *k,* respectively.

It is assumed that $w_{k}$ and $v_{k}$ are independent:

(13) $$\{\begin{array}{c} w_{k}∼N(0,Q)\\ v_{k}∼N(0,R) \end{array}$$

As we all know, the purpose of attacks against ICPS is to downgrade a stable operation state to a target state [21] and to evade detection, which could cause some indicators to deviate from the normal range. Different studies choose different standard indexes to identify anomalies, such as 2-normal, Chi-square, cost function, etc.

Although they are different methods, they are essentially all based on related errors. References [8,22,23] use a Chi-square detector to detect a CPS abnormity if the error between an estimated value and the real value exceeds a threshold. Although this method is unable to detect false data-injection attacks, it is applicable for other types of attack, for example: DoS attacks. The error covariance between the state value and estimated value, which we call minimum mean-square error (MMSE), is used in [24] to detect abnormal actions caused by network attacks because DoS attacks will break the system balance, which will increase system regulation cost. References [2,21] adopt a cost function to judge whether a system is under attack or not. 

In many studies, a Kalman filter was utilized to perform state estimation, distinguish deviations and detect mistakes from observations under malicious attacks. In our research, we formulate a cost function that penalizes deviations from normal to abnormal states, and detects whether a DoS attack has happened. In this section, we model the physical part of an ICPS as a time-varying linear control system, which is equipped with a Kalman filter, LQR controller and failure detector:

(14) $$\{\begin{array}{c} \begin{array}{c} {\tilde{x}}_{k+1|k}=A{\tilde{x}}_{k}+Bu_{k}\;\\ P_{k+1|k}=AP_{k}A^{T}+Q \end{array}\\ K_{k}=P_{k|k-1}C^{T}{\left[CP_{k|k-1}C^{T}+R\right]}^{-1}\\ P_{k}=P_{k|k-1}-K_{k}CP_{k|k-1}\\ {\tilde{x}}_{k+1}={\tilde{x}}_{k+1|k}+K_{k}[y_{k}-C{\tilde{x}}_{k+1|k}] \end{array}$$

where ${\tilde{x}}_{k}$ is the a posteriori state estimation value at time k, the error ($x_{k}-{\tilde{x}}_{k+1|k}$) is between the estimation and real value, $P_{k}=\mathrm{cov}(x_{k}-{\tilde{x}}_{k})$ is the error covariance that shows the accuracy of the a priori estimation, $A^{T}$ is the transposed matrix of A, and $X_{k=0}=X_{0}$, $P_{k=0}=P_{0}$.

According to [23,25], although the gain of Kalman filter *K_k_* is time-varying, it always converges in a few steps to guarantee the system is detectable, so it can be defined as follows:

(15) $$K≜K_{k}=P_{k|k-1}C^{T}{\left[CP_{k|k-1}C^{T}+R\right]}^{-1}$$

At the same time, in order to simplify the analysis, it is usually assumed that the initial state of an ICPS with a linear state feedback controller is stable. Based on the LQR controller used in control systems, we assumed it is used in the ICPS to minimize the cost function, and the usefulness of the controller is to minimize the cost function *J’* as much as possible as follows:

(16) $$J≜\min\underset{T\to \infty }{\lim}E\frac{1}{T}\left[\sum_{k=0}^{T-1}\left(e_{k}^{T}We_{k}+u_{k}^{T}Uu_{k}\right)\right]$$

where $e_{k}=x_{k}-{\tilde{x}}_{k}$ and the matrices of *W* and *U* are assumed as positive semi-definite matrices. 

When an ICPS is attacked by a DoS attack, the attacker’s intention is to transfer the running zone $\Omega_{1}$ to other zones which will certainly increase the time delay. The increase of time delay means increasing the cost function, which also adds to the system’s operation cost. Obviously, the communication time delay located in the network layer caused by a DoS attack will increase the cost function of the control system located in the physical layer. That is to say, the purpose of a malicious attacker is to degrade the stable running state of the control system of the physical layer by attacking the network layer, so Problem 3.1 can be rewritten as:

**Problem 4.1 (Attacker’s Purpose):**  (17) $$\{\begin{array}{l} \max\;J\\ s.t.\;I_{H}=1 \end{array}$$

*It uses a Kalman filter to provide a system optimal state estimate of*${\tilde{x}}_{k}$, so it can be obtained that:

(18) $${\tilde{x}}_{k}={\tilde{x}}_{k|k-1}+K[y_{k}-C{\tilde{x}}_{k|k-1}]$$

*and we can get the optimal control law of LQR with fixed gain:*

(19) $$u_{k}=-{\left(B^{T}SB+U\right)}^{-1}B^{T}SA{\tilde{x}}_{k}$$

*where matrix S satisfies the Riccati equation:*

(20) $$S=A^{T}SA+W+A^{T}SB{\left(B^{T}SB+U\right)}^{-1}B^{T}SA$$

*If we want to keep the ICPS running stably, we must make sure both J and the error are not unbounded. That is to say, it can be determined whether there is a DoS attack from whether the cost function J is bounded.*

*Simultaneously, it also defines a threshold function*$J_{th}$*J_th_*:

(21) $$J_{th}≜\max\underset{T\to \infty }{\lim}E\frac{1}{T}\left[\sum_{k=0}^{T-1}\left(e_{k}^{T}We_{k}+u_{k}^{T}Uu_{k}\right)\right]$$

*so the detector works successfully with the following condition:*

(22) $$\{\begin{array}{l} J>J_{th}\;,\;alarm\\ J\leq J_{th}\;,\;no\;alarm \end{array}$$

*This can trigger an attack alarm under DoS attack when the cost function exceeds the threshold.*

### 4.2. Mimicry Security of Defense Policy

**Figure 8:**
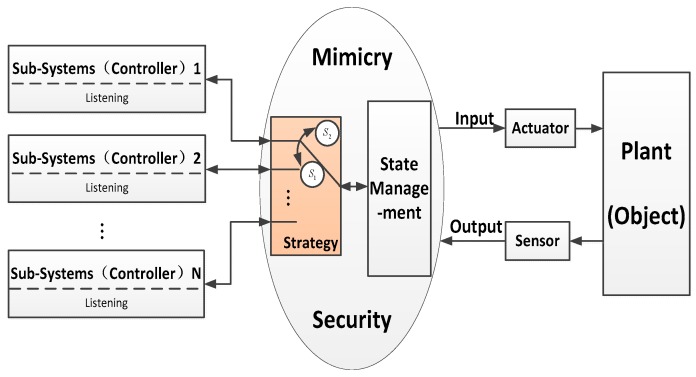
Mimicry security defense strategy.

According to the DoS attack model, when an ICPS with one sub-system running is under attack, it could increase the time delay or even the rise of control cost as depicted in Problem 3.1 or Problem 4.1. In this sub-section, a mimicry security method is presented to solve this problem, which includes a state management and a mimicry switch strategy. It requires every sub-system to be waiting for running in real time. The state management is listening to all sub-systems’ running states, inputs, outputs, detection of DoS attacks and other running state variables. The mimicry switch strategy is responsible for switching the running sub-system equipped with different network configurations and same control algorithm on the basis of switch rules from the attack detection of state management, which is depicted in the following Figure 8. 

From Subsection 3.3, it is known that the ICPS is secure when it is running in $\Omega_{1}$. That means we can keep the ICPS running normally, whether the defense strategy is used or not. Only when the ICPS is running in the $\Omega_{2}$ state under attack, we must adopt the mimicry defense strategy in time, keep the physical part stable, and ensure it is not operating in $\Omega_{3}$, so the function of the defense strategy is to solve Problem 3.1 (or Problem 4.1) which can be rewritten as the following Problem 4.2 under the condition of the mimicry transformation time $T_{\sigma (\cdot )}$:

**Problem 4.2**  (23) $$\{\begin{array}{l} g_{\sigma (•)}(o_{i})\\ s.t.\;I_{H}=1\\ s.t.\;T_{\sigma (•)} \end{array}$$

*According to Equation (10), when*$I_{H}=1$*, which means*$\Omega_{ICPS}\in (\Omega_{2}\cup \Omega_{3})$*Combined with Remark 2, we know*$\Omega_{ICPS}\in \Omega_{2}$. $∵P(\Omega_{ICPS}\in \Omega_{2}|I_{H}=1)=P(\Omega_{ICPS}\in \Omega_{2})$*, so Equation (23) becomes:*

(24) $$\{\begin{array}{c} g_{\sigma (•)}(o_{i})\\ s.t.\;(\Omega_{ICPS}\in \Omega_{2})\\ s.t.\;T_{\sigma (•)} \end{array}$$

$∵\sup\;T_{ICPS}=\tau_{1}$$∴\inf\;(\tau_{2}-T_{ICPS})=\tau_{2}-\tau_{1}=\tau$*. Combined with equations (1) and (2), Equation (24) becomes:*

(25) $$\{\begin{array}{l} f_{\sigma (t)}\;(t)\\ \{\begin{array}{l} \dot{x}(t)=A_{\sigma (t)}x(t)+B_{\sigma (t)}u(t)\\ y(t)=C_{\sigma (t)}x(t) \end{array}\\ s.t.\;(\Omega_{ICPS}\in \Omega_{2})\\ s.t.\;T_{\sigma (\cdot )}\leq \tau \end{array}$$

From the above analysis, we know a new sub-system with new network configuration is waiting to run. Based on Equation (4), once the ICPS adopted a mimicry security to defend a DoS attack, the cyber part can work normally immediately, which will make the attack useless by transferring the ICPS to the $\Omega_{1}$ from the $\Omega_{2}$. 

As is known to us, the sub-systems of ICPSs are changed after eliminating the impact of the cyber layer. However, if we want to protect the whole ICPS, we must keep the balance of the physical part. Hence, the problem that the mimicry security strategy needs to solve is changed from Problem 4.2 to Problem 4.3:

**Problem 4.3**  (26) $$\{\begin{array}{l} \dot{x}(t)=A_{\sigma (t)}x(t)+B_{\sigma (t)}u(t)\\ y(t)=C_{\sigma (t)}x(t)\\ s.t.\;T_{\sigma (•)}\leq \tau \\ \sigma (•):J\geq J_{th} \end{array}$$

If we want to make the ICPS free from paralysis, we should not only eliminate adverse effects from the network part, but also ensure the physical plant keeps running normally. When we adopt mimicry security strategy to switch sub-systems with different cyber parameters, it makes the system’s cyber part be free from malicious actions instantly, so we need to design a switching controller to guarantee the every sub-system is running after switching.

Combined with equations (1), (2), (11) and (22), the switching signal $\sigma (•):J\geq J_{th}$ can be converted into $\sigma (•):\underset{T\to \infty }{\lim}E\frac{1}{T}\left[\sum_{k=0}^{T-1}\left(e_{k}^{T}We_{k}+u_{k}^{T}Uu_{k}\right)\right]\geq J_{th}$. 

Define a switching sequence: $\left\{x_{k};i_{1},i_{2},\cdots ,i_{k},\cdots |i_{k}\in N,k=0,1....\right\}$. Therefore, the physical plant with feedback gain is:

(27) $$\{\begin{array}{l} \dot{x}(t)=A_{\sigma (t)}x(t)+B_{\sigma (t)}u(t)\\ y(t)=C_{\sigma (t)}x(t)\\ s.t.\;T_{\sigma (\cdot )}\leq \tau \\ \sigma (•):J\geq J_{th} \end{array}$$

To keep the ICPS running normally, it is also needed to make *J* ≤ *J_th_* after using the mimicry switch strategy, so the solution to Problem 4.3 becomes how to design a switching feedback gain *K* = {*K_i_* | *i* = 0,1....,*N*}. If we want to keep the new sub-system stable after a mimicry switch, we need a positive definite matrix *P* [26,27]. At the same time, according to Theorem 1 in paper [28], the system must satisfy a bound to achieve a guaranteed cost function:

(28) $$\{\begin{array}{c} A_{i}^{T}PA_{i}-P+Q+K_{i}^{T}RK_{i}<0\\ J\leq X_{0}^{T}PX_{0} \end{array}$$

**Proof of Equation 28.**  Considering a known definite matrix P, and a Lyapunov function V(x(k)),

Make $V(x(k))=x{\left(k\right)}^{T}Px(k)=\sum_{i=1}^{N}V_{i}(x(k))$

Obviously: V(x(k))=0 only x(k)=0; and V(x(k))>0 when x(k)≠0. Then:

$$\begin{array}{ll} \Delta V(x(k))= & x{\left(k+1\right)}^{T}Px(k+1)-x{\left(k\right)}^{T}Px(k)\\ & =\sum_{i=1}^{N}\Delta V_{i}(x(k))=\sum_{i=1}^{N}\left(V_{i}(x(k+1))-V_{i}(x(k))\right)\\ & =\sum_{i=1}^{N}\left[{\left((A_{i}+B_{i}K_{i})x(k)\right)}^{T}P((A_{i}+B_{i}K_{i})x(k))-x{\left(k\right)}^{T}Px(k)\right]\\ & =\sum_{i=1}^{N}\left[{\left({\bar{A}}_{i}x(k)\right)}^{T}P({\bar{A}}_{i}x(k))-x{\left(k\right)}^{T}Px(k)\right]\\ & =\sum_{i=1}^{N}\left[x{\left(k\right)}^{T}{\bar{A}}_{i}^{T}P{\bar{A}}_{i}x(k)-x{\left(k\right)}^{T}Px(k)\right]\\ & =\sum_{i=1}^{N}\left[x{\left(k\right)}^{T}({\bar{A}}_{i}^{T}P{\bar{A}}_{i}-P)x(k)\right] \end{array}$$

From (28): ΔV(x(k))<0.

## 5. Experiments

**Figure 9:**
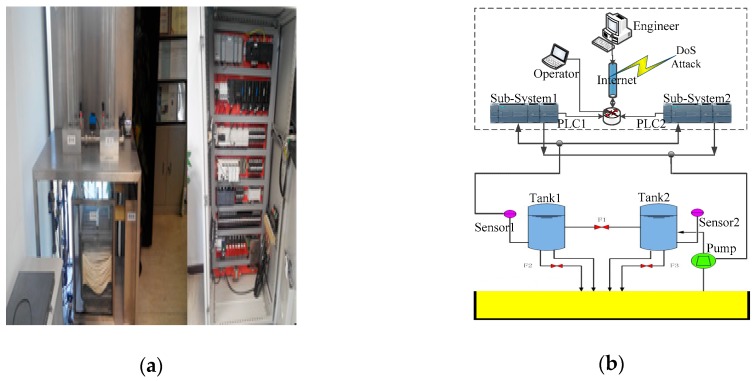
This is the platform used in our experiments. (**a**) Test Platform Entity; (**b**) Test Platform Framework.

In this section, we do some experiments to test our algorithms on a platform as depicted in Figure 8 which used a real industrial control system equipped with some industrial computers, sensors, electric motors, programmable logic controllers, a network server, cloud server and so on. Besides, this typical ICPS communicated by a network as shown in Figure 9.

On this experimental platform, we use two Siemens’ programmable logic controllers (PLCs), which were set to two different IP addresses and two communication ports. The workflow of this platform is that the pump will pump some water into Tank 2 and keep a certain liquid level when the valve *F*_1_ between Tank 1 and Tank 2 and the valve *F*_2_ are opened. In our paper, we use the state of liquid level *H* that is the system response as an indicator to show whether the ICPS is being attacked by a DoS attack. The steady state of this liquid level *H* and sampling time are set as 400 mm and 0.2 s, respectively.

### 5.1. Related Network Feature

**Figure 10:**
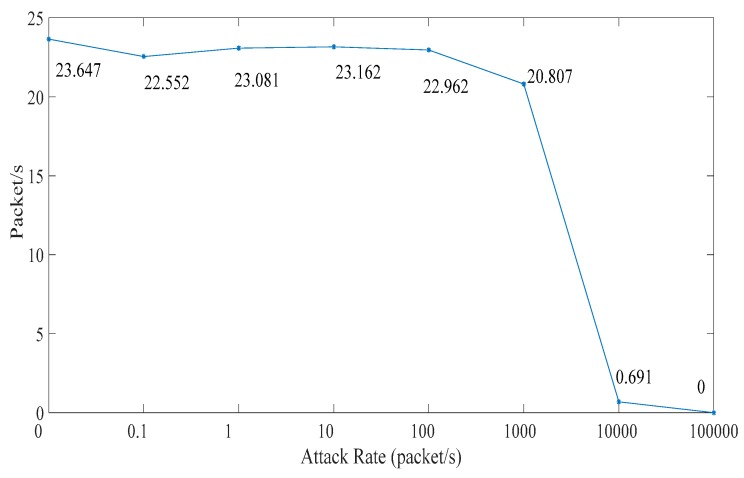
Test packet number per-second under different DoS attack rates.

**Figure 11:**
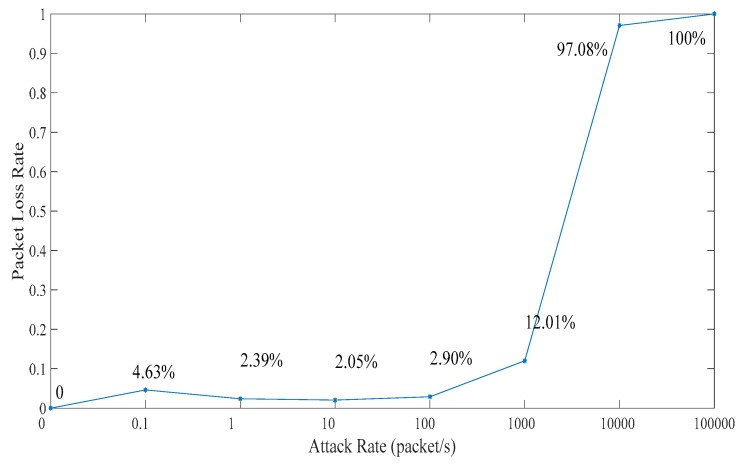
Test packet loss rate under different DoS attack rates.

**Figure 12:**
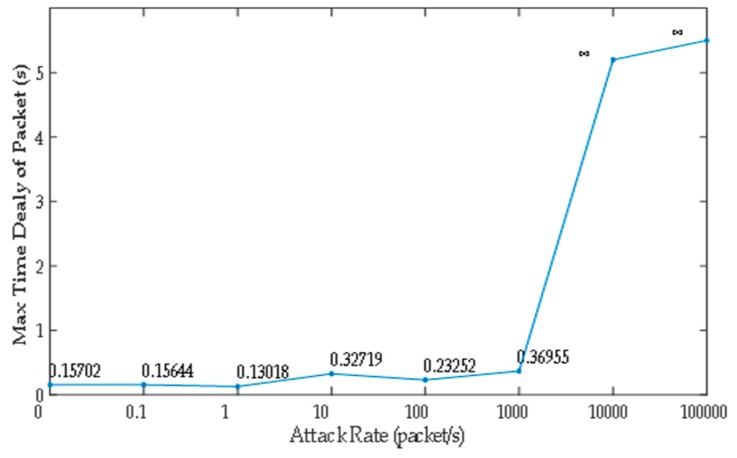
Max transmission time delay under different DoS attack rates.

**Figure 13:**
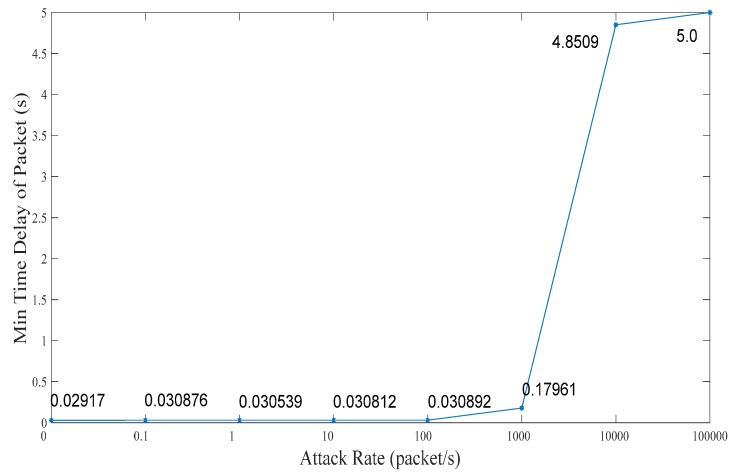
Min transmission time delay under different DoS attack rates.

**Figure 14:**
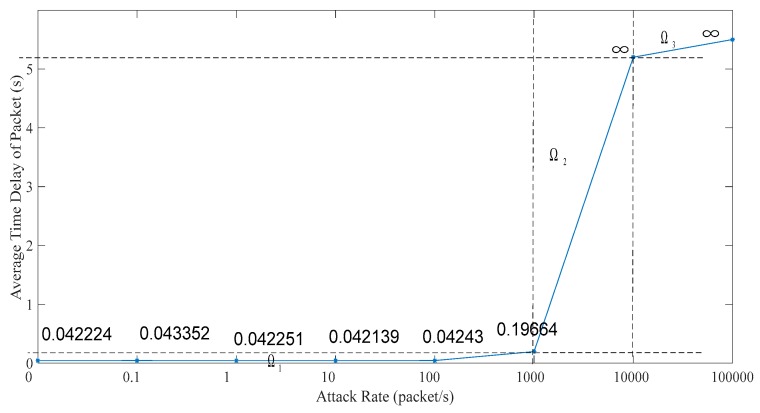
Average transmission time delay under different DoS attack rates.

In this subsection, we analyze the effect on the ICPS’s cyber part caused by DoS attacks with different attack rates. We repeat the DoS attacks against the PLC controller of ICPS 100 by the Monte Carlo method to get details of the network features used in the communication network. Firstly, we designed a probe to test the communication time delay between the PLC controller and the upper computer located in the Alibaba cloud server. Secondly, we used the hping3 network tool to implement DoS attacks with different attack rates for at least 1 minute each time. Then, we made use of the probe to randomly test the time delay for 60 to 600 s. What’s more, this experiment was repeated 100 times. Finally, it the statistical data was achieved and the relation between attack rate and time delay (TD) were obtained, as displayed in Table 1 and Figure 10, Figure 11, Figure 12, Figure 13 and Figure 14. 

Actually, the configuration software (for example: Intouch) used in industrial control systems always has a default time delay (5 s, 10 s, or 15 s and so on), which means once the time delay of data packets from the sender exceeds a default value, the system will trigger an alarm. In our paper, we set 5 s as a default value, which is the time delay threshold.

On this experimental platform, the PLC controllers send data to the upper computer and receive data from the upper computer. A 5 s socket timeout was designed, which means that if new data was not sent or new data was not accepted for more than five seconds, the communication connection was considered broken. In Table 1, ∞ indicates that the PLC controller’s network has crashed due to a high attack DoS attack rate.

The number of test packets from the probe is relatively stable when the attack rate is less than 1000. However, the change is sharply reduced when the attack rate is more than 1000 as seen in Figure 10. It can be seen that the packet loss rate is opposite to the above test packets numbers from the probe in Figure 11. When the attack rate is more than 1000, the packet loss rate will sharply increase until no packet data exists.

We can get that the maximum, minimum and average time delay of ICPS’s network from Figure 12, Figure 13 and Figure 14, respectively. No matter which the time delay it, its data trend is basically the same. They all reflect that the communication delay will increase with the rise of DoS attack rates until the network services has crashed undoubtedly, but every kind of time delay has only a little change when that attack rate is less than 1000. 

The above table and charts show that the normal running of ICPS will not be affected by malicious attacks when the attack rate is less than a certain value. Usually, if the ICPS is not attacked by a DoS attack or the ICPS is under the DoS attack with an attack rate lower than *fr*_2_, the time delay must be in the range Min TD to Max TD. Due to the performance limitations of network devices, the time delay can’t be less than Min TD. Once the time delay exceeds Max TD, the performance of the ICPS will be destroyed. The randomness of time delay makes us select Average TD as an indicator to show the network performance. That is to say, we don’t have to consider DoS attacks when the ICPS is running in the $\Omega_{1}$ zone, which also demonstrates the correctness of Section 4.2.

### 5.2. Mimicry Security Strategy

In this subsection, we analyze the effect on an ICPS’s physical part against DoS attack with different attack rates. The liquid level *H* (system response) and cost function of ICPS are illustrated in detail here, when it is in stable status.

**Figure 15:**
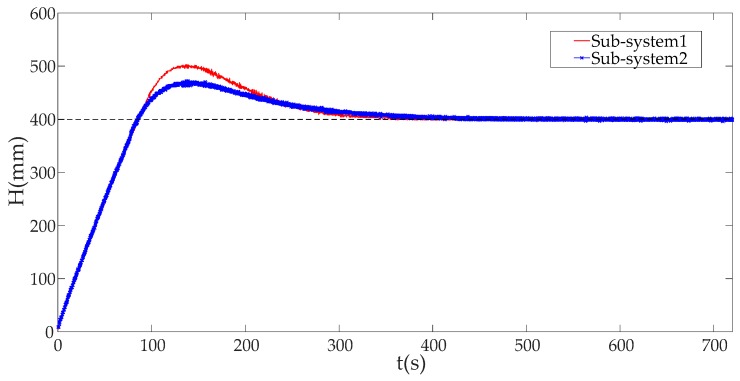
The system responses with different sub-system without DoS attack.

Figure 15 depicts the system response without a DoS attack. We can see that no matter which sub-system was used, the ICPS whose sub-systems had different IP addresses and ports could stabilize the liquid level of platform at the same height *H* = 400 mm without a DoS attack.

**Figure 16:**
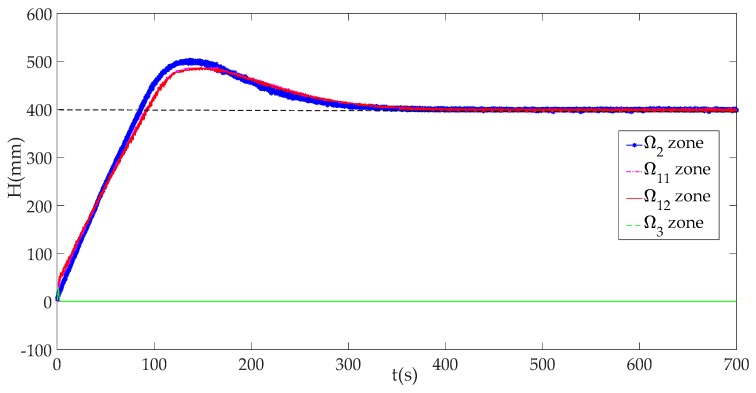
System responses in different zones.

Figure 16 shows that system can keep running normally in the $\Omega_{1}$ zone (including $\Omega_{11}$ and $\Omega_{12}$) and $\Omega_{2}$, but not in the $\Omega_{3}$ zone. That is to say, the physical plant will not function well once that DoS attack rate exceeds a certain value. This has proved the validity of security zones. 

Combining Figure 10, Figure 11, Figure 12, Figure 13, Figure 14, Figure 15 and Figure 16, we can conclude that this ICPS is not affected by DoS attacks within a certain range of attack rates. However, once the DoS attack rate is more than a threshold, this malicious action will seriously damage the natural communication function of the cyber part, and will also affect the normal operation of the physical plant seriously in turn.

**Figure 17:**
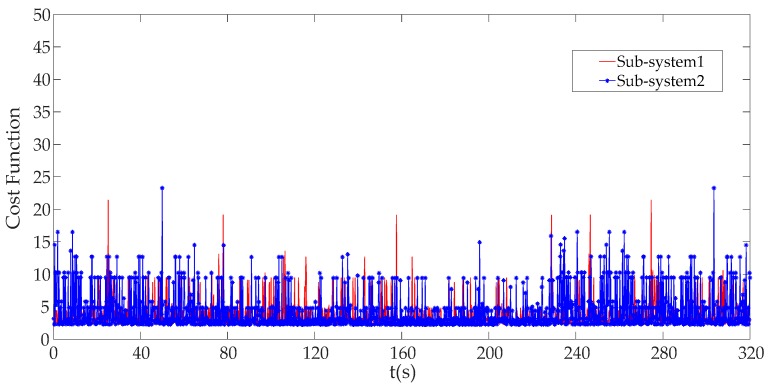
The cost function without DoS attack.

**Figure 18:**
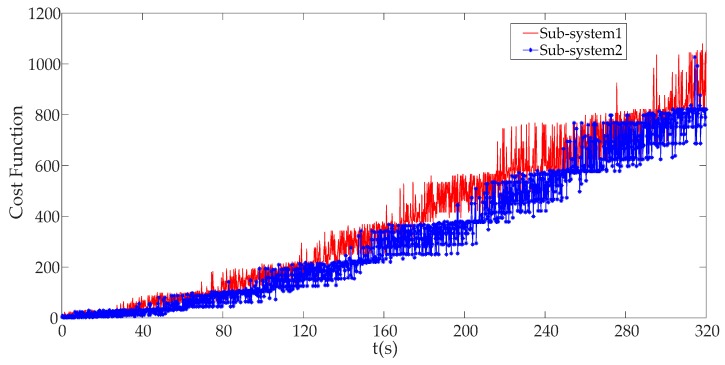
The cost function under DoS attack with an attack rate of 1000.

We can see that the cost function value is very small and relatively stable in the stable running state of the control system in Figure 17, but, it will increase sharply under DoS attack with an attack rate of 1000 as shown in Figure 18.

Comparing Figure 17 with Figure 18, it can be seen that the cost function value *J* of ICPS will be enlarged more times. Obviously, once the DoS attack is launched by malicious attackers, we must have *J* > *J_th_*, which will trigger an alarm.

**Figure 19:**
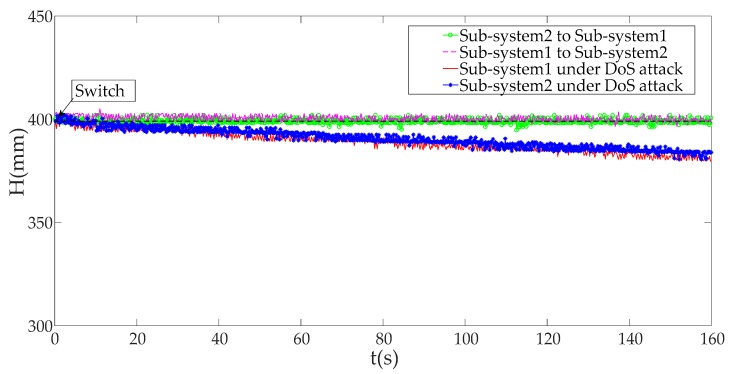
Different system responses before and after using the switch strategy.

When an alarm is triggered, the mimicry security strategy will be used to protect the ICPS against the DoS attack. In our paper, we take sub-system 1 and sub-system 2 as an example. When the ICPS is under a DoS attack with an attack rate greater than 1000, the liquid tank level *H* controlled by the physical plant with sub-system1 begins to become unstable, and the same situation happens to sub-system2; However, when we use the mimicry security switch strategy to switch sub-system 2 with IP2 and port 2, the original ICPS equipped with sub-system 1 with IP1 and port1 become stable again, which is the same as switching ICPS’s sub-system 2 to sub-system 1 as displayed in Figure 19.

There is no doubt that the mimicry security strategy can solve the DoS attack against ICPS, which proves that this defense strategy is effective.

### 5.3. Results Comparison

**Figure 20:**
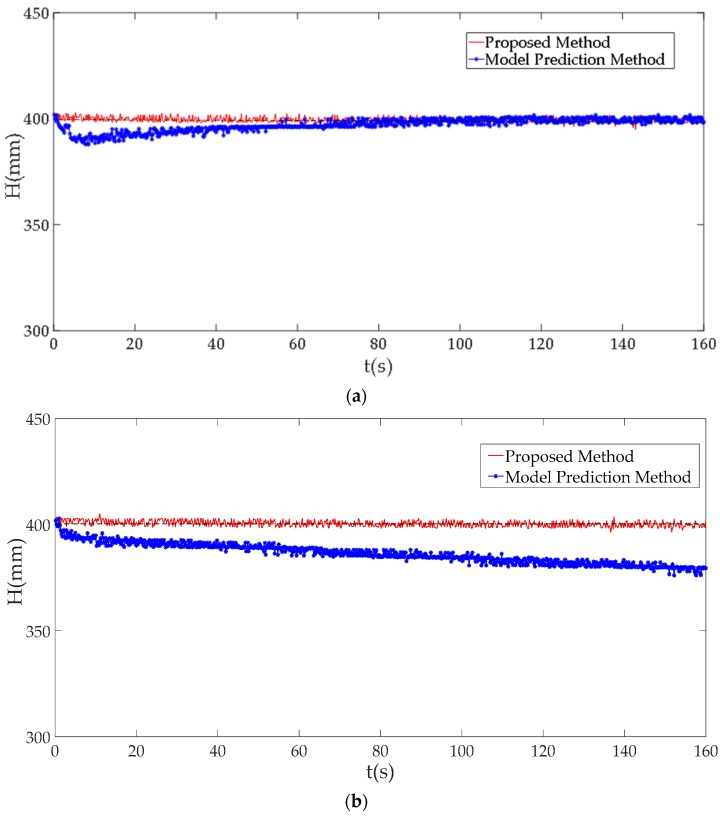
The comparison of results using different methods. (**a**) Experimental results under a DoS attack rate of 1000; (**b**) Experimental results under a DoS attack rate of 10,000.

In this subsection, we compare with some experimental results using different methods to show details about the effectiveness of method proposed by us. In our paper, a predicted model-based algorithm [29,30] is selected as a contrast to show the usefulness of our proposed method. Figure 20 shows the comparisons of experimental results under different DoS attack rates. Obviously, both our method and the predicted method can eliminate the impacts on the physical plant caused by DoS attacks under the attack a rate of 1000 as is shown in Figure 20a. However, when the ICPS is under DoS attack with a rate of 10,000, our method can still work to keep the operation stable, while the method based on model prediction is invalid.

**Figure 21:**
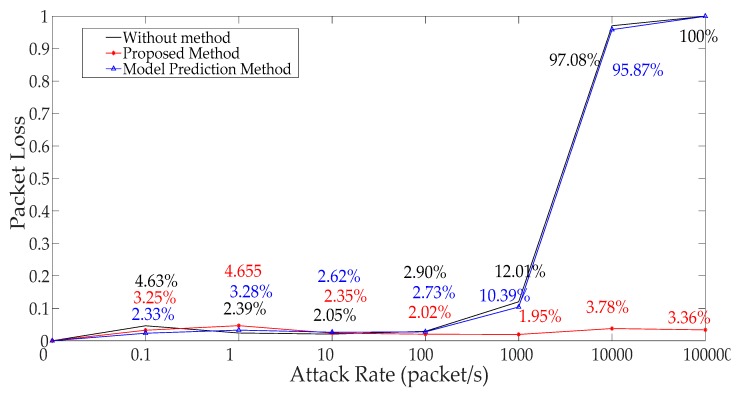
Different packet losses using different methods.

Figure 21 shows that the comparisons of different packet loss of experimental results when we use different methods. It is obvious that this malicious attack can’t affect the packet loss when the ICPS was under a DoS attack with an attack rate of less than 1000, which is equivalent to saying that that the ICPS is running in $\Omega_{1}$. However, when the attack rate is more than 1000 ($\Omega_{2}\cup \Omega_{3}$), and our method can solve this serious network problem through switching to a new sub-system equipped with a new IP address and communication port. The method based on model prediction does not have this function.

**Figure 22:**
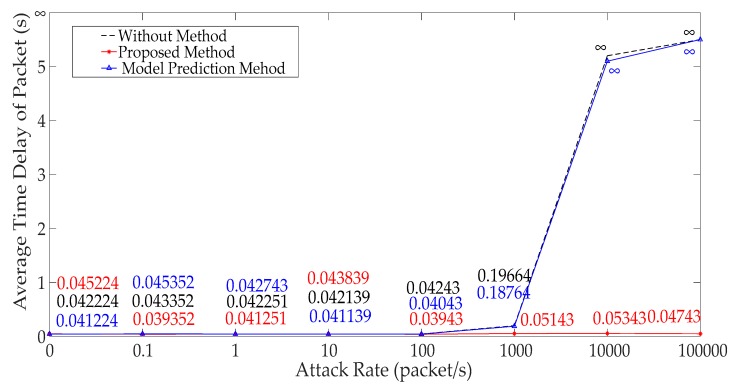
Different time delays using different methods.

Figure 22 depicts the comparison of different average time delays of experimental results when we use the different methods. It can be obtained that our method can deal with the huge time delays caused by the DoS attack when the attack rate is more than 1000 and less than 10,000 ($\Omega_{2}$), especially the controller crash problem when the attack rate is more than 10,000 ($\Omega_{3}$), but, the predicted method based on the model cannot clear up it.

From Figure 20, Figure 21 and Figure 22, we know that the method proposed by us not only solves the network problems caused by DoS attacks, but also can maintain the normal operation of the ICPS. The algorithm based on model prediction is still unable to handle the cyber problem caused by this DoS attack with great attack rates. Even the physical process can’t remain stable when the attack rate is too large ($\Omega_{2}$ ∪ $\Omega_{3}$).

Obviously due to the resilient control ability of network devices, some DoS attacks with low attack rates cannot affect the normal operation of an ICPS. Previous works will produce some false alarms and reduce the detection accuracy, because of the existing resilient ability of network devices. Most models built by previous works don’t consider specific DoS attacks, which is equivalent to studying the situation of ICPS running in zones $\Omega_{1}$, $\Omega_{2}$ and $\Omega_{3}$. However, our paper takes this robustness of network devices into consideration and it is simplified further by studying their operation conditions in different security zones. That is to say, we don’t need the DoS attacks of $\Omega_{1}$, so all we have to deal with is the DoS attacks of $\Omega_{2}$ and $\Omega_{3}$, which need to be detected no matter which method is used. When the DoS attacks which belong to zones of $\Omega_{2}$ and $\Omega_{3}$ are launched, our method not only detects this malicious action, but also maintains the physical process stable and eliminates the serious impact on the cyber layer, while the method based on model prediction cannot deal with the two aspects of the problem at the same time.

## 6. Conclusions

In this paper, we study DoS attack problems and build related mathematic models to explain how DoS attacks affect the stable operation of ICPSs with different attack rates, which are based on studying the impacts of attacks on the cyber part and physical plant, respectively.

According to different attack rates, we divide them into different running zones firstly, which is consistent with facts. Then, we build a DoS attack model and explain the effect on an ICPS against attack actions using the above zones instead of analyzing the time delay from ICPSs’ control data directly, which also shows clearly that the ICPS has a defense ability against malicious DoS attacks. The time delays ought to be negligible and the impact is fatal once the DoS attack rate exceeds a threshold. What’s more, we chose the cost function value as a norm to detect anomalous actions and propose a mimicry security switch strategy to defend against such malicious attacks. Finally, we modeled a lot of DoS attacks and used a mimicry switch strategy repeatedly. From the above table and charts we can obviously see the impacts on the ICPS’s cyber part and physical plant caused by this malicious action. The comparisons with different experimental results also verify our model’s correctness and our method’s effectiveness.

## Figures and Tables

**Table 1:** The time delay caused by different DoS attack rates.

Attack Rate	0	0.1	1	10	100	1000	10000	100000
Min TD (ms)	29.170	30.876	30.539	30.812	30.892	179.614	4850.917	5000
Max TD (ms)	157.017	156.437	130.180	327.192	232.517	369.547
Average TD (ms)	42.224	43.352	42.251	42.139	42.430	196.638	∞
Average Packet/s	23.647	22.552	23.081	23.162	22.962	20.807	0.691	0
